# Assessing Heat Stress and Health among Construction Workers in a Changing Climate: A Review

**DOI:** 10.3390/ijerph15020247

**Published:** 2018-02-01

**Authors:** Payel Acharya, Bethany Boggess, Kai Zhang

**Affiliations:** 1 Department of Epidemiology, Human Genetics and Environmental Sciences, School of Public Health, The University of Texas Health Science Center at Houston, Houston, TX 77030, USA; pacharya06@gmail.com (P.A.); bcboggess@gmail.com (B.B.); 2 Workers Defense Project, Austin, TX 78753, USA; 3 Southwest Center for Occupational and Environmental Health, School of Public Health, The University of Texas Health Science Center at Houston, Houston, TX 77030, USA

**Keywords:** climate change, construction workers, heat-related illness, heat stress

## Abstract

Construction workers are at an elevated risk of heat stress, due to the strenuous nature of the work, high temperature work condition, and a changing climate. An increasing number of workers are at risk, as the industry’s growth has been fueled by high demand and vast numbers of immigrant workers entering into the U.S., the Middle East and Asia to meet the demand. The risk of heat-related illnesses is increased by the fact that little to no regulations are present and/or enforced to protect these workers. This review recognizes the issues by summarizing epidemiological studies both in the U.S. and internationally. These studies have assessed the severity with which construction workers are affected by heat stress, risk factors and co-morbidities associated with heat-related illnesses in the construction industry, vulnerable populations, and efforts in implementing preventive measures.

## 1. Introduction

Heat stress poses a substantial risk to construction workers worldwide in a changing climate. Construction workers are vulnerable to heat stress because the majority (e.g., 73% in the U.S.) [1] engage in heavy work outdoors. Construction workers in the southern United States, the Middle East, Asia, Latin America, and Africa are regularly exposed to extremely high temperatures with long working hours, yet may have limited or no access to shade or water [2]. Previous studies have shown that construction workers in the U.S. are 13 times more likely to die from a heat-related illness (HRI) compared to workers in other industries, and within the industry, roofers and road construction workers face a particularly high risk of HRIs [3,4].

Projected increases in extreme heat due to changing climate, along with other factors, are expected to increase the vulnerability of construction workers to heat stress [2]. The global construction industry generates 12% of the world’s gross domestic product and is expected to grow rapidly as populations in China, southern Asia, and the U.S. continue to expand [5]. As construction workers comprise an increasingly large and critical part of the global economy, special attention needs to be paid to the risks faced by the global construction workforce from occupational heat stress. In addition, greenhouse gas emissions are increasing mainly driven by human activities, and the scientific community has a consensus that climate change is taking place with a general trend of rising temperatures [6]. As a result, the number of extreme hot days are projected to last longer with more frequency and intensity in the future [7]. Additional factors such as long working hours compounded by heavy workloads, impediments in regulating small construction businesses, and vulnerabilities due to an immigrant worker status that limits access to healthcare resources further worsen construction workers’ vulnerability to heat stress.

In this paper, we will first review the existing epidemiological research about occupational heat stress in the construction industry, both in the U.S. and internationally. Then we investigate the risk factors for heat-related illnesses in construction, as well as populations most vulnerable to experiencing heat-related illness. Additionally, we review preventive measures and existing worker protection policies enacted by local and national governments.

## 2. Method

We conducted a general scoping review [8] to identify the scientific papers published and other relevant information available, including governmental and non-governmental organization (NGO) reports. The databases that were searched included PubMed, Web of Science, references of relevant peer-reviewed literature, and web-based searches (including Google Scholar and documents published by occupational-health organizations). Key words and phrases that were used to search the databases included “construction workers”, “heat stress”, “heat-related illness”, “construction industry”, “work rest cycles”, “regulations” and derivatives of the words “heat”, “temperature”, “hot”, “morbidity”, “risk factors”, “mortality”, “injury”. Due to the limited number of scientific articles available, the search was broad and was not limited by study type. However, studies published after March 2017 were not included. For sources other than scientific literature, reports and conference papers from governmental, non-governmental and inter-governmental organizations were included. Material written in languages other than English were not included (*N* = 2). Search results that addressed or analyzed heat stress exposure, risk of HRIs, or interventions for HRIs among construction workers were selected for this review.

## 3. Results

### 3.1. Types of Metrics for Heat Exposure

Heat stress depends on many variables such as temperature, humidity, wind, clothing, shade, physical activities, and other factors. These factors can vary greatly depending on the environment, the occupation, and the individual worker. Workers felt more uncomfortable in a hot and humid environment than in a hot and dry environment, with heat stress further exacerbated by heavy workload, personal protective equipment (PPE), among other factors [9]. Therefore, a variety of measures have been used to characterize heat stress, including simple temperature metrics (e.g., daily maximum or minimum temperatures), composite indices accounting for temperature and other weather parameters such as humidity (e.g., heat index), core body temperature, and skin temperature. This is mainly due to the fact that heat stress is the heat load brought about by many factors such as weather conditions, physical activities, metabolic heat and thermal effects of clothing [10,11]. Wet Bulb Globe Temperature (WBGT) is a commonly used measure in occupational settings that incorporates air temperature, humidity, radiant heat and wind speed [11]. The WBGT also serves as the metric upon which heat stress standard ISO 7243 (International Organization for Standardization) for determining ergonomic effects of thermal environments is based [12], and it is a widely used heat index that underlies measurement of the Threshold Limit Values (TLVs) by the American Conference of Governmental and Industrial Hygienists (ACGIH) [10]. Despite limitations of WBGT in measuring the effects of metabolic rate and effect of wind speed [12], WBGT is still an important index to measure heat effects. Another index of heat stress relevant to construction workers and workers laboring in the outdoors is the Thermal Work Limit (TWL), a commonly used measure in occupational settings that incorporates environmental parameters into single index as the equivalent metabolic rate [11]. Although core body temperature and skin temperature are better measures than other indicators of environmental heat, they have not been used very often because of safety and logistical issues and because they require individuals to ingest or attach a temperature sensor [13]. Several studies in environmental epidemiology that have examined the associations between heat and mortality concluded that the ‘best measure to heat stress varied with populations and regions’ [9]. Other heat indices are the Humidex (used in Canada) and the National Weather Service (NWS) Heat Index in the U.S., both describing how hot the weather feels by combining the effects of heat and humidity [11].

### 3.2. Epidemiological Studies

Table 1 summarized 16 epidemiological studies included in this review, including study design, study location/period, study population, heat exposure metrics, outcomes and major conclusions.

#### 3.2.1. Construction Industry is Severely Affected by Heat Stress

According to the study by Xiang et al. (2014) [14], workers in the construction industry are one of the most affected by heat stress, second only to agricultural workers. The percentage change in daily injury claims per °C increase in daily maximum temperature (T_max_) below the threshold temperature (37.7 °C) resulted in an incidence rate ratio (IRR) of 1.006 (95% confidence interval CI: 1.002–1.011). Washington State Accepted State Fund (SF) Workers’ Compensation data from 1995 to 2005 showed that the construction industry was responsible for 33.1% of all HRI claims, and that the construction industry had the highest HRI claim rate (12.1 per 100,000 Full-time Equivalent, FTE) [3]. Research conducted in Taiwan between 2004 and 2007 by Lin and Chan (2009) [15] found that the perceived risk of excessive heat in the workplace was highest among the workers from the construction industry. A small study of 16 rebar workers in Beijing found that labor productivity decreased with increasing temperatures, and that older or less-experienced workers had greater productivity losses [16].

Among studies investigating heat-related mortality outcomes among workers, it was found that the construction industry had consistently higher fatality rates related to heat stress as compared to other industries. For example, in a case-control study conducted in the state of Arizona in the U.S., the odds of heat-related mortality were highest among construction/extraction workers (*N* = 76; OR = 2.32; 95% CI 1.55–3.48, age-adjusted) [17]. In the U.S., the construction industry accounted for 36.8% of the occupational heat-related mortality nationwide. The U.S. construction workers, over a 10-year period, had 13 times (RR = 13.0; 95% CI 10.1–16.7) higher risk of a heat-related fatality compared to workers in other industries [4]. Sett and Sahu (2014) [18] studied the effects of heat exposure on female brick workers in India, where construction is heavily reliant on bricks, and it was found that the weekly productivity among those workers declined under increased exposure to outdoor heat, and their physiological stress parameters such as peak heart rate and cardiac strain, as measured by Net Cardiac Cost and Relative Cardiac Cost, were significantly higher in elevated temperatures. 

#### 3.2.2. Pattern of Heat Stress Injuries

The number of daily HRIs increase as the ambient temperature increases beyond a certain range. The morbidity effects of rising temperatures are slow at first, but they rise steadily as the temperature continues to increase [19]. In the study conducted by Xiang et al. (2014) [14] in Adelaide, Australia, an inverse U-shaped relationship was found between T_max_ and the number of workers’ injury claims reported between July 2001 and June 2010. Until the threshold temperature of up to 37.7 °C, ambient temperature was positively associated with the risk of work injury for all the outdoor industries, including the construction industry, whereas a negative association was observed between the workers’ injuries and temperature beyond this point. This may be due to workers halting work at higher temperatures and thus leading to small sample size of reported claims. The threshold temperature was determined by choosing a single cut-off point from the range of recorded temperatures using the hockey-stick model. A threshold temperature of 37.7 °C was ascertained and associations between temperature and daily injury claims were quantified above and below the threshold temperature.

### 3.3. Policies, Regulations, and Recommendations

Few regulations exist to prevent HRIs in the construction industry, even though construction workers are among the most likely to experience them. Heat-related illnesses and fatalities are easily prevented with appropriate rest, shade, and rehydration. The U.S. Occupational Safety and Health Administration (OSHA) (2011) [20] has published guideline for employers, and it recommend that preventive activities increase as the heat index increases. In brief, according to the range of heat index values, OSHA defined four risk categories: Lower; Moderate; High; and Very High/Extreme. Recommendations include providing rest, shade and water; training; acclimatization; developing a monitor system for HRI signs; limiting physical tasks; rescheduling non-essential work; and closely monitoring workers’ vital signs and strictly enforcing work/rest cycles, and the choice of these recommendations depends on risk categories. It is also important to note that working in direct sunlight or unventilated buildings can increase the ambient temperature to a greater degree. Suggestions have been made by a number of agencies, including the ACGIH and the UK Health and Safety Executive (HSE), regarding upper limit of heart rate. The ACGIH recommends upper limit of 120 beats per minute for one-minute recovery heart rate, whereas the HSE suggests the heart rate threshold at workplace to be calculated from the age of individual workers. Further, ISO 7933 sets limits for body mass loss as a measurement of heat strain as 5% for 95% of the working population [10,11].

#### 3.3.1. Public Education Campaigns and Governmental Guidelines

Few construction workers globally are protected from the risks of occupational HRIs by enforceable policies, but many countries have implemented educational campaigns. Some countries have issued strong recommendations for employers, but these recommendations are generally not enforceable. The European Union, the U.S., India, and many other countries recommend that employers follow such guidelines, but they do not enforce them strictly [21]. Some municipalities, such as Ahmedabad in Gujarat, India, have acted to prevent HRIs by issuing high temperature warnings and distributing educational pamphlets to the public on heat stress prevention [22,23]. However, the efficacy of such guidelines or public education campaigns to prevent or reduce HRIs among construction workers is unknown.

#### 3.3.2. Limited Work Hours

Qatar, the United Arab Emirates, and other Middle Eastern countries limit work hours in summer by requiring all work to stop between 11:30 a.m. and 3:00 p.m. [24]. However, this regulation is irregularly enforced and temperatures can still be extremely high during non-restricted hours. Investigations by the Amnesty International [25] found that migratory construction workers were still laboring during limited work hours in Qatar. Nepalese and Indian embassies have reported dozens of young men who have died from heart attacks triggered by heat stress while working in the Middle East [25], indicating that the regulatory standard is ineffective. The national guidelines for heat stress management in China delineated in the document entitled “Notice for administering guidelines on climatic heat stress prevention measures” were issued in 2012, and regulate work hours based on environmental threshold of forecasted daily maximum air temperature [26]. Information about the efficacy of these regulations was not found.

#### 3.3.3. Required Rest Breaks

In 2015, Costa Rica introduced legislation requiring employers of agricultural workers who labor outdoors to provide shade, water, rest breaks, and protective clothing [27]. The legislation is modeled on the US, OSHA guidelines for protecting workers at various heat index levels, with increased protections as the heat index increases [20]. It was created in response to a growing epidemic of chronic kidney disease linked to occupational heat stress and chronic dehydration among young men in Costa Rica, Nicaragua and El Salvador [28]. Although this legislation was not specifically applied to construction workers, globally this is the only comprehensive, enforceable legislation implemented at a national level to protect a subset of outdoor workers from HRIs. While other countries, such as the United Arab Emirates (UAE), have adopted policies to protect outdoor workers from heat stress, these policies may be insufficient or poorly enforced. For example, in 2005 the Ministry of Labor of UAE banned work between 12:30 and 4:30 p.m. in July and August, but the duration was restricted a year later to 12:30 to 3:00 p.m. from lobbying by construction companies [29].

The state of California in the United States [30] requires employers to provide rest, shade and potable water to agricultural workers, and when temperatures exceed 95 °F (35 °C) monitoring for signs of HRIs must be conducted by designated co-workers or a supervisor. Agricultural workers must receive at least ten minutes of rest every two hours when temperatures are at or above 95 °F (35 °C); however, no other outdoor workers are included in this provision. In 2010 and 2015, the municipalities of Austin [31] and Dallas [32], Texas in the U.S. adopted mandatory rest breaks for construction workers of at least ten minutes for every four hours of work. Heat indices often average over 110 °F in these cities from May to September. Prior to the enactment of these regulations, repeated surveys of Texas construction workers found that nearly 40% of them were unable to take rest or water breaks during those days [33].

### 3.4. Risk Factors

#### 3.4.1. Physiological Effects of Heat Stress

HRIs occurs when the body retains more heat than it can release, which can then lead to a range of symptoms including heat stroke and death. The effects of heat stress were classified according to the International Classification of Diseases, Ninth Revision, Clinical Modification (ICD-9-CM) that is used to assign codes following hospital utilization in the United States [34]: 992.0—heat stroke and sunstroke; 992.1—heat syncope; 992.2—heat cramps; 992.3—heat exhaustion, anhydrotic; 992.4—heat exhaustion due to salt depletion; 992.5—heat exhaustion, unspecified; 992.6—heat fatigue, transient; 992.7—heat edema; 992.8—other specified heat effects; or 992.9—effects of heat and light, unspecified; and/or an ANSI Z16.2 type code 151 (contact with general heat—atmosphere or environment) [3]. In ambient heat at or above 34–37 °C (93–99 °F), the only method of heat loss from the body is through evaporation of sweat. Accompanied by high humidity, sweat evaporation is greatly reduced resulting in the rising of the core body temperature to potentially dangerous levels (>39 °C or 102 °F) [35,36]. Kjellstrom (2016) [35] reports many such direct impacts of heat on workers performing heavy labor, such as in the construction industry, including mortality due to cardiovascular conditions. Additionally, in labor-intensive and outdoor occupations, increased environmental heat has been found to be associated with chronic kidney disease, teratogenic effects and poor clinical status, in addition to reduced work performance resulting in loss of income.

Heat stress can also lead to issues with a worker’s hydration status. Hydration status is dependent upon factors such as perspiration rate, the amount of water intake [37], working conditions, choices such as clothing types [38], and personal behaviors such as alcohol consumption [39]. Urine Specific Gravity (USG) is considered as a good indicator of absolute hydration status of the body. The maximum concentrating capacity of the renal system is 1.050, as represented by ascertainment of acutely dehydrated state at Urine Specific Gravity (USG) value of >1.030. On the other hand, a USG value of <1.015 is considered an euhydrated state [40]. In a comparative study aimed at measuring hydration status among construction workers working under sunlight versus non-exposed conditions in Iran, a significant correlation was found between thermal work limit (TWL) and the USG measures. The exposed group of workers had significantly higher (*p* value 0.001) mean USG level (1.026 ± 0.005) than the non-exposed group (mean USG of 1.021 ± 0.005), indicating a hypo-hydrated to clinically dehydrated status in the heat-exposed group [41]. Contrarily, in a study conducted by Bates (2008) [42] in the United Arab Emirates, the construction workers studied were not found to have dehydrated status (average water intake was 5.44 liters per 12-h shift). However, in another study conducted by Bates et al. (2010) [43] among expatriate construction workers working in outdoor and/or in significant heat-generating industrial conditions in the Middle East, the workers were hypo-hydrated as measured by Urine Specific Gravity (USG) values that were elevated during midday and afternoon [3]. Additional discrepancies in assessing extent of heat stress is addressed by Basagana (2014) [19] who commented that time-series studies on mortality and morbidity due to heat stress often do not take into consideration other external causes of outcomes.

#### 3.4.2. Seasonal Aspect of HRIs

According to Bonauto (2007) [3], most of the HRI claims (*N* = 456; 95% of total claims) across all the industries in Washington State of the U.S. were made between May through September, which were the area’s hottest months. Similarly, a vast majority of days that recorded multiple HRI claims were between June and August. Among all the HRI claims made in the third quarter of July–September, the construction industry remained highest in terms of number (%) of HRI claims at 121 out of 351 claims (34.5%).

#### 3.4.3. Co-Morbid Risk Factors for Heat-Related Stress

A number of co-morbid risk factors for HRIs have been identified in the literature. According to Bonauto et al. (2007) [3], use of medications, illicit drugs or alcohol were seen in about 22% of the HRI claims, and other physiological co-morbid conditions to HRIs may arise due to lack/loss of sleep, fatigue and disease. A multiple regression analysis showed that the rate of perceived exertion (RPE) of the construction workers was related to alcohol consumption (standardized coefficient = 0.62, rank = 1), as was duration of work and smoking habit. Also, among construction workers in Hong Kong, smokers represented a higher rate of heat disorder cases (17.8%) than non-smokers (15.2%) [44]. However, as mentioned by the authors, a lack of set standard of drug use history and medical record collection among workers indicate that these data simply represent a crude estimate, and similar caution should be exercised in interpreting the regression analysis rankings. Body Mass Index (BMI) measurements of obesity (21.4%) and underweight (30.0%) groups had higher percentage of HRI cases [44]. Institutional factors contributing to HRIs have also been identified at the ecosystem level (weather and climate), society level (e.g., policy and culture), industry level (e.g., workers’ training), organization level (e.g., business model), and individual level (e.g., work skills, risk perception) [45].

#### 3.4.4. Business Size and Skill Levels

According to Xiang et al. (2014) [14] who studied workers’ compensation claims in Adelaide, Australia between 2001 and 2010, business size was found to be inversely associated with daily injury claims of the workers. Injury claims increased by 0.7% (IRR = 1.007, 95% CI 1.003–1.011) for small businesses and 0.4% (IRR = 1.004, 95% CI 1.002–1.006) for medium-sized businesses per 1 °C increase in maximum temperature below threshold (37.7 °C). Moreover, in the study conducted by Bates et al. (2010) [43] among expatriate workers in hot conditions in the Middle East, it was found that among the different working conditions and skill levels studied, the unskilled and semi-skilled workers (island development and city development) had higher USG compared to skilled tradesmen, indicating poorer hydration status [43].

#### 3.4.5. Vulnerable Age Ranges

Varying results were identified from the literature regarding vulnerable age groups. For example, Xiang et al. (2014) [14] found that in Adelaide, Australia, young male workers across all industries were at most risk of heat stress, where the incidence rate ratios (IRRs) for male workers and young workers aged ≤24 were IRR = 1.004 (95% CI 1.002–1.006) and IRR = 1.005 (95% CI 1.002–1.008), respectively. In Washington State in the U.S., Bonauto (2007) [3] found the most vulnerable age group for HRI claims as 25–34, closely followed by 18–24 and 35–44-year-old male workers. It can be concluded from the paper that the workers between ages 18 and 44 comprised the group with the most HRI claims (about 75% of the claims). Interestingly, as the temperature exceeded T_max_ the injury claims decreased, except for the age group 55 years and older. Similar findings were reported by Jia et al. (2016) [44] where the highest percentage of HRI cases were found among construction workers in the 26 to 35-year-old group (23.7%). The number of cases decreased with age.

Older workers may also be at higher risk of experiencing HRIs and heat-related mortalities. In the state of Arizona, U.S., Petitti et al. (2013) [17] found that the proportion of heat-related mortality was higher for workers aged 35–49 (112, 25.2% of cases) and 50–65 (114, 25.7% of cases) among construction and agricultural workers. Similar to this finding, occupational heat-related mortality data from the Bureau of Labor Statistics (BLS) in the US between 2000 and 2010 shows that mortality among workers aged 35–54 accounted for 53% (27.3% for ages 35–44; 25.9% for ages 45–54) of all heat-related mortalities. Among the older U.S. workers (ages ≥ 65 years) the average rate of occupational heat-related fatalities per million workers per year was found to be the higher at 0.32, versus that of workers <55 years at 0.22 [34]. Xiang et al. (2014) [14] found that in Australia, the injury claims of the ≥55 years age-group continued to increase when the T_max_ exceeded threshold temperature, contrary to the other age groups, where injuries decreased beyond the threshold temperature.

#### 3.4.6. Sex and Racial Differences

Differences in heat stress risk by sex and race among construction workers is not well documented in the literature, but such disparities have been documented among workers in other industries. Among U.S. workers in all industries, Hispanic men were found to have significantly higher age-adjusted odds ratio for heat-related mortality (OR = 2.69; 95% CI 1.79–4.05), along with Native-American men (OR = 2.43; 95% CI 1.79–4.05), compared to non-Hispanic white male workers. A similar trend was observed in female workers when non-Hispanic white women were used as the reference group (Hispanic women; OR = 2.79; 95% CI 1.56–7.0), but an even higher odds ratio for heat-related deaths was observed among Native-American female workers (OR = 3.81; 95% CI 1.51–9.57) [17]. A number of studies have reported significantly lower number of HRI claims among female workers compared to male workers [3,14,17]. For every degree rise in temperature below the threshold temperature of 37.7 °C, daily HRI claims by male workers in Australia increased by 0.4% (IRR 1.004, 95% CI 1.002–1.006). However, no such effect was observed by the study among the female workers whose IRR for both above and below threshold temperature remained insignificant (*p* value of 0.550 and 0.206) [14]. Jia et al. (2016) [44] reported a similar finding in their study among construction workers in Hong Kong where men had a higher number of heat disorder cases than females (out of 36 HRI cases, 35 were male workers). The authors commented that the result might be due to lighter workload of females than the males in the construction industry [44]. The Bureau of Labor Statistics (BLS) data for the U.S. heat-related mortality between 2000 and 2010 found that Blacks were at elevated risk of heat-related occupational mortality than the non-Hispanic Whites (RR = 1.5; 95% CI 1.1–2.0). Also, Hispanics were found to be at higher risk (RR = 3.2; 95% CI 2.5–4.0) compared to non-Hispanics. Hispanic workers also had a significantly high average yearly HRI mortality rate of 0.54 per 1 million workers [44]. Overall, racial minorities were found to have a higher risk of heat-related mortality in the U.S.

## 4. Prevention

### 4.1. Effects of Acclimatization to Heat

Human beings subjected to repeated exposure to hot environments over a period of time undergo physiological responses to heat changes such as an increased and quicker onset of sweating in response to increased heat. This phenomenon is termed as acclimatization, which is characterized by increased blood volume, a decrease in internal body temperature, and a decrease in sodium chloride content of sweat and urine, along with better coping of hot conditions as internal body temperature and heart rate remain within acceptable limits in response to heat stress. Lack of acclimatization has been attributed to heatstroke, heat syncope, heat exhaustion and heat cramps [36].

Bonauto et al. (2007) [3] suggests that workers who were not physiologically well adjusted to a high workplace ambient temperature and higher exertion levels had greater heat-related stress. In this study, the author used the ‘length of time employed’ as a measure of acclimatization. For all workers’ claims, it was observed that within a period of one week or less of employment, workers sustained higher HRIs (14%), as compared to other general health-related claims (3.3%). Poor acclimatization was also reflected by the fact that regardless of the length of employment (considered as a measure of acclimatization), a sudden and significant increase in daily maximum temperature (of 10 °F or 5.5 °C) was associated with approximately 42% of the HRI claims. Morioka et al. (2006) [37] points out that among construction workers, physiological adaptation to heat stress begins within three to four days of working in hot conditions but the hormonal regulation process of acclimatization starts three to four weeks later. This delayed response may mean that the workers are at an increased risk for experiencing HRIs if heat stress prevention measures are not provided or adequately used.

### 4.2. Optimizing Work–Rest Cycles

Heat strain can be reduced by regular and frequent periods of rest when workers are experiencing heat stress. Yi and Chan (2013) [46] used Monte Carlo simulation to estimate the probability distribution of physiological conditions and behavioral factors (e.g., heart rate, blood pressure, percentage of body fat, and smoking habits) and environmental conditions (e.g., WBGT, air pollution index (API) of rebar workers) to calculate optimum break schedule. It was found that a 15-min break after working constantly for 120 min (WBGT = 28.9 ± 1.3 °C) would be optimum. A slightly increased rest period and lowered working period were recommended. The Physiological Strain Index (PSI), which is based on heart rate and core temperature and is used to measure heat strain during exercise, was used to measure the rates of recovery after heat stress in 19 rebar workers. It was found that 94% of the recovery occurred within 40 min of rest, 84% in 20 min, and 58% in 5 min, when workers were allowed to work to exhaustion [47]. Further, based on Time-Weighted Average (TWA), recovery time for heavy workload has been summarized by Rowlinson and Jia (2014) [13] as follows:

- 28.5 °C-WBGT: 120 min of work followed by a 5-min break
- 28.9 °C-WBGT: 90 min of work followed by a 10-min break
- 29.7 °C-WBGT: 60 min of work followed by a 15-min break
- 31.6 °C-WBGT: Self pace

For ordinary work, self-paced work was suggested from temperatures of 31.7 °C-WBGT or higher, up to the sustainable work limit of 240 min [13]. These suggestions may be used for designing work–rest cycles. The continuous work time (CWT) reference values were, in turn, calculated from the maximum allowable exposure duration (D_lim_) [11].

### 4.3. Other Preventive Actions

A number of workplace recommendations have been made by the National Institute for Occupational Safety and Health (NIOSH) for hot environments, including engineering controls and heat alert program [36]. In a study on Japanese construction workers, Morioka et al. (2006) [37] and [36] has found that preventive heat stress measures like the provision of electric fans for ventilation, cool water dispensers, ice machines and structured rest periods were crucial in reducing heat stress. Proper breakfast and electrolyte supplements are recommended as well.

## 5. Climate Change

According to the Intergovernmental Panel on Climate Change’s Fifth Assessment Report, greenhouse gas emissions have been increasing since the pre-industrial era and are mainly driven by human activities. The scientific community has a consensus that climate change is taking place with a general trend of increasing temperatures [48]. Available weather observations indicate that the period 1983–2012 ranked the highest in the 30-year period in the Northern Hemisphere, and the linear slope of the global average surface temperature was calculated as 0.85 °C during the period 1880–2012. In fact, growing evidence has suggested that greenhouse gas emissions attributed to anthropogenic activities account for more than half of the increase in the globally averaged surface temperature during the period 1951–2010 [48].

As a consequence of the changing climate, heat waves are projected to last longer and occur more frequently and intensely [7]. This has been generally confirmed by many studies using a variety of climate models and scenarios. Moreover, the trend of increasing heat wave days in many regions in recent years is in the agreement of climate change projections [49]. Globally, the heat wave in Europe in 2003 caused 14,802 deaths in France alone and the heat waves in the future were estimated to occur at least twice as frequently as the 2003 European heat waves [50]. The chance of a 2010 Russian heat wave that was associated with 55,000 deaths is estimated to become 5 to 10 times more likely by 2050 [49,51]. In the U.S., high summertime temperatures and heat waves are projected to increase in most regions, particularly in the western and central U.S. [52]. If greenhouse gas emissions continue to grow globally, the hottest 5% of the summertime temperatures during the period 1950–1979 are projected to occur at least 70% of the time in 2035–2064; and the chance of previous once-in-20-year heat wave days are projected to happen up to 10 times in most of the US in the late 21st century [52].

## 6. Conclusions

In summary, heat-related health effects among construction workers are a significant but understudied public health topic. This is a critical omission given the trend of the generally increasing global temperatures. Adverse heat-related health effects can be reduced readily through low-cost interventions (e.g., more breaks and the provision of shade and drinking water). Lundgren et al. (2013) [53] summarized the research needs for all working populations in regard to climate change. The latest assessment of the impacts of climate change on human health [7] also highlights research needs for general populations. Some of these research needs are particularly appropriate and important for construction workers. They include: (1) the role of genetic and epigenetic factors and social determinants in developing heat-related health effects; (2) exposure–response associations under a large range of temperatures and across locations; (3) the combined effects of heat stress and other stressors (e.g., air pollution): and (4) developing more effective intervention and prevention action plans.

## Figures and Tables

**Table 1:** Summary of heat-related epidemiological studies among construction workers.

First Author	Study Location/Study Period	Sample Population	Study Design/Data Source	Heat Exposure Metric	Health Outcomes	Main Conclusions
Bonauto et al. 2007 [3]	Washington, United States 1995–2005	Workers’ compensation claims (*N* = 480)	Ecological study Heat-related Ilness (HRI) worker compensation claims	Temperature	Heat-related illness (HRI)	Construction industry had highest HRI claims in terms of percentage of HRI claims (33.1%), number of outdoor claims (146 out of 377) and claim rate (12.1 per 100,000 Full-time Equivalent (FTE)) Higher temperatures (daily maximum temperature (T_max_) average of 88.5 °F) led to multiple HRI claims compared to single claims Age groups 18–44 claimed most (75%) HRI injuries
Gubernot et al. 2015 [4]	United States 2000–2010	Heat-related deaths for workers (*N* = 359)	Retrospective study Census of Fatal Occupational Injuries database of Bureau of Labor Statistics	-	Heat-related mortality	Construction industry had 13 times (rate ratio, RR = 13.0; 95% confidence interval CI 10.1–16.7) more heat-related fatality compared to other industries, and overall highest percentage of HRI deaths (36.8%) Mortality among workers aged 35–54 accounted for 53% of all heat-related mortalities. Older workers (ages ≥ 65 years) had higher rate of heat-related fatalities compared to younger workers (0.32 versus 0.22, respectively, per million workers per year) Highest percentage of heat-related mortality (86%) occurred in summer (June–August), majority of workers (70%) dying on the day of exposure Higher heat-related mortality among Hispanics (RR = 3.2; 95% CI 2.5–4.0), Blacks (RR = 1.5; 95% CI 1.1–2.0), when compared to Whites
Rowlinson and Jia 2014 [13]	Hong Kong June–September 2011	Construction workers (*N* = 216)	Cross-sectional study Participants in the study	Wet Bulb Globe Temperature (WBGT)	Heart rate (beats per minute)	For heavy work, no compulsory rest needed below 28.3 °C WBGT For ordinary work, continuous work time sufficient to exclude recovery period; self-pace work recommended for 31.7 °C WBGT
Xiang et al. 2014 [14]	Adelaide, Australia July 2001–June 2010	Workers’ compensation claims (*N* = 252,183)	Retrospective study SafeWork South Australia injury claim data	T_max_	Work-related injuries	Construction industry ranked among the highest for daily injury claims (Incidence rate ratio, IRR = 1.006; 95% CI: 1.002–1.011) Inversed U-shaped relationship between daily maximum temperature and daily mean injury claims (T_max_ at 37.7 °C) Daily injury claims for male workers and young workers (IRR = 1.004, 95% CI: 1.002–1.006) and IRR = 1.005 (95% CI: 1.002–1.008), respectively aged ≤ 24 were highest. Injury claims of the ≥55-year age group continued to increase beyond T_max_ Business size inversely associated with daily injury claims: per 1 °C increase in maximum temperature injury claims increased by 0.7% (IRR = 1.007, 95% CI: 1.003–1.011) for small- and 0.4% (IRR = 1.004, 95% CI: 1.002–1.006) for medium-sized businesses
Lin and Chan 2009 [15]	Taiwan 2001–2007	Workers’ records from a variety of industries including construction (*N* = 10,403,000; all industries combined)	Retrospective study Publicly available Taiwanese government database	WBGT	Perceived a risk of excessive heat	Construction industry has highest percentage of perceived risk of excessive heat (76.3%) at workplace Construction industry comprises younger and middle-aged workers while Agriculture/Forestry/Fishing industry comprised more elderly workers Hot season (May-October) with average maximum temperature > 30 °C and relative humidity > 74% pose health threats for workers
Petitti et al. 2013 [17]	Maricopa County, Arizona, US 2002–2009	Heat-caused deaths (Cases *N* = 444 Control *N* = 925)	Case-control study Death certificates	-	Heat-related deaths	Percentage of heat-related mortality was highest among construction/extraction workers (*N* = 76; OR = 2.32; 95% CI 1.55–3.48) Proportion of heat-related mortality was higher for workers aged 35–49 (112, 25.2% of cases) and 50–65 (114, 25.7% of cases) among construction workers Hispanic men had significantly higher age-adjusted odds ratio for heat-related mortality (OR = 2.69; 95% CI 1.79–4.05), along with Native-American men (OR = 2.43; 95% CI 1.79–4.05), compared to non-Hispanic white male workers. Similar trend was observed in Hispanic women (OR = 2.79; 95% CI 1.56–7.0) and Native-American women (OR = 3.81; 95% CI 1.51–9.57)
Sett and Sahu 2014 [18]	West Bengal, India October 2008–May 2009, October 2009–May 2010, and October 2010–May 2011	Female brick workers (*N* = 120)	Questionnaire Participants in the study	WBGT	Cardiac parameters (peak heart rate, net cardiac cost, relative cardiac cost, and recovery heart rates)	Linear decline in productivity with increased maximum air temperature above 34.9 °C Net cardiac cost, recovery- and peak- heart rates significantly higher on hotter days (WBGT outdoor index: 26.9 °C to 30.7 °C) than on cooler days (WBGT outdoor index: 16.1 °C to 19.3 °C)
Morioka et al. 2006 [37]	Wakayama Prefecture, Japan August 1998	Construction workers (*N* = 12 male workers)	Cross-sectional study Participants in the study	WBGT	Health problems as measured by blood urea nitrogen (BUN), blood sugar, serum osmotic pressure	Blood sugar before work (103.4 ± 15.5 mg/dL) significantly higher than after work (93.0 ± 10.5 mg/dL) Unaltered BUN and serum electrolytes during work suggests breakfast was effective in replenishing salinity Preventive heat-stress measures (ventilation, cool water and structured rest periods) crucial to reduce heat stress.
Chan et al. 2013 [38]	Hong Kong July–September 2010	Rebar workers aged 20–60 years (*N* = 10)	Prospective study Participants in the study	Thermal Work Limit (TWL)	Ratings of perceived exertion (RPE)	Environmental factors causing increase in RPE include duration of work, air pollution; personal factors include age, alcohol and smoking habits
Inaba and Mirbod 2007 [39]	Gifu city, Japan August 2001	Traffic control workers (*N* = 247); Male workers engaged in building construction (*N* = 115)	Questionnaire Participants in the study	WBGT	Heat prevention measures in summer (self-reported symptoms classified in categories of frequency)	Prevalence of alcohol intake in construction workers (45.2%) greater than that of traffic-control workers (24%) Overall, construction workers had significantly higher musculoskeletal and general heat-related symptoms during summer than traffic-control workers
Montazer et al. 2013 [41]	Iran Date not provided	Sun-exposed and non-exposed construction workers (*N* = 60)	Cross-sectional study Participants in the study	WBGT, TWL	Hydration status (measured by urine specific gravity, USG)	Exposed group of workers had significantly higher mean USG level (1.026 ± 0.005) than the non-exposed group (mean USG of 1.0213 ± 0.0054), indicating a hypo-hydrated to clinically dehydrated status in the heat-exposed group Pearson correlation coefficients showed a significant correlation of −0.93 between USG and TWL Exposed group of workers had significantly higher mean USG level (1.026 ± 0.005) than the non-exposed group (mean USG of 1.0213 ± 0.0054), indicating a hypo-hydrated to clinically dehydrated status in the heat-exposed group Pearson correlation coefficients showed a significant correlation of −0.93 between USG and TWL
Bates and Schneider 2008 [42]	Al Ain, United Arab Emirates May 2006	Construction workers (*N* = 22)	Cross-sectional study Participants in the study	WBGT, TWL	Hydration status and physiological workload- as measured by aural temperature, fluid intake, and USG	USG <1.015, indicating “well-hydrated” workers. Average fluid intake was 5.44 liters per 12-h shift per day
Bates et al. 2010 [43]	Abu Dhabi and Dubai, United Arab Emirates Sites 1 and 2 in July and August; sites 3 and 4 in September and December 2009, respectively	Expatriate workers (manual laborers) in construction and other industries (*N* = 186)	Cross-sectional study Participants in the study	-	USG	Unskilled and semi-skilled workers had higher USG (1.020 ± 0.008) compared to skilled tradesmen (USG = 1.016 ± 0.009), indicating poorer hydration status among the former group
Ji et al. 2016 [44]	Hong Kong 2011	Construction workers (*N* = 216)	Ecologic study HRI cases	Temperature, humidity, solar radiant heat, WBGT	HRI	Smokers represented higher rate of heart disorder cases (17.0%) than non-smokers (15.2%) Underweight (30.0%) and obesity (21.4%) groups had higher heat-related illness cases
Yi and Chan 2013 [46]	Hong Kong July 2010 –September 2011	Rebar workers (*N* = 29)	Prospective study Participants in the study	WBGT	Heat tolerance time (HTT)	Optimized schedule of having a 15-min break after working 120 min continuously in the morning (WBGT = 28.9 ±1.3 °C), and having a 20-min break after working 115 min continuously in the afternoon (WBGT = 32.1 ± 2.1 °C) is proposed by the authors
Chan et al. 2012 [47]	Hong Kong July–August 2011	Rebar workers (*N* = 19)	Cross-sectional study Participants in the study	WBGT	Recovery time measured by Physiological Strain Index (PSI); RPE	On average, a rebar worker could achieve 94% recovery in 40 min; 93% in 35 min; 92% in 30 min; 88% in 25 min; 84% in 20 min; 78% in 15 min; 68% in 10 min; and 58% in 5 min; recovery time is a significant variable to predict rate of recovery (*R*^2^ = 0.99, *p* < 0.05)
